# The epidemiology of cancer in Angola—results from the cancer registry of the national oncology centre of Luanda, Angola

**DOI:** 10.3332/ecancer.2015.510

**Published:** 2015-02-17

**Authors:** António Armando, Mary Clarisse Bozzetti, Alice de Medeiros Zelmanowicz, Fernando Miguel

**Affiliations:** 1 Postgraduate Program of Epidemiology, Department of Social Medicine, School of Medicine, Universidade Federal do Rio Grande do Sul (UFRGS), Rua Ramiro Barcelos, 2400, 2º andar, 90035-003 – Porto Alegre, RS, Brazil; 2 National Oncology Centre of Luanda, Rua Amilcar Cabral, s/n, Junto Ao Hospital Pediátrico António Bernardino-Maianga, Luanda, Angola; 3 Universidade Federal de Ciências da Saúde de Porto Alegre (UFCSPA), Brotherhood of the Santa Casa de Misericórdia of Porto Alegre (ISCMPA), Rua Professor Annes Dias, 295, 90020-090 – Porto Alegre, RS, Brazil

**Keywords:** Angola, cancer, epidemiology

## Abstract

Knowledge of the epidemiological profile of cancer is a key step in planning national cancer policy. The main objective of this study was to characterize the epidemiological profile of cancer in Angola based on cases of cancer registered at the National Oncology Centre (NOC) of Luanda, the only Angolan hospital to specialize in cancer treatment and diagnosis. The study consisted of a cross-sectional historical review of cases treated at the NOC between 2007 and 2011. The following variables were analysed: tumour location, diagnostic basis, and source of referral, as well as patient age, sex, place of residence, and the stage of the disease. The NOC registered a total of 4,791 patients throughout the study period, at an annual average of 958 cases. The most commonly diagnosed cancers were breast (20.5%), cervical (16.5%), and head and neck cancer (10.6%), followed by lymphoma (7.2%), Kaposi sarcoma (6.1%), and prostate cancer (4%). A total of 76% of patients were under 60 years old, and 10% were less than 15 years old. Of the total number of patients with cancer treated at the NOC, 77.3% lived in the Luanda province. Staging data were only available for patients with breast or cervical cancer, and an analysis of this variable showed that most of these individuals were in advanced stages of the disease. In the absence of a population-based cancer registry, this study constitutes a reasonable assessment of the epidemiological profile of cancer in Angola.

## Introduction

Cancer is a worldwide public health issue and has severe social and economic repercussions. According to the International Agency for Research on Cancer (IARC), a total of 14.1 million new cases of the disease and 8.2 million cancer-related deaths were reported in 2012 [1]. Projections based on these results indicate that the worldwide incidence of cancer will reach approximately 19.3 million by the year 2020 [1, 2, 3].

In the light of these findings, the World Health Organization (WHO) has recommended that all countries develop cancer control programmes to reduce the incidence and mortality associated with this disease [4]. However, the successful planning, implementation, and evaluation of cancer control programmes require the availability of epidemiological data regarding the frequency and the distribution of cancer in each region provided by a population-based cancer registry (PBCR) [4, 5].

The absence of PBCR in Angola has led to a lack of knowledge of the epidemiology of the disease and represents a limitation in the development of a successful national cancer policy [2, 6]. According to the IARC, in this situation, hospital-based cancer registries in large oncology centres may give very important information, providing a reasonable estimate of the epidemiological profile of cancer in the population [4].

**Figure 1.**
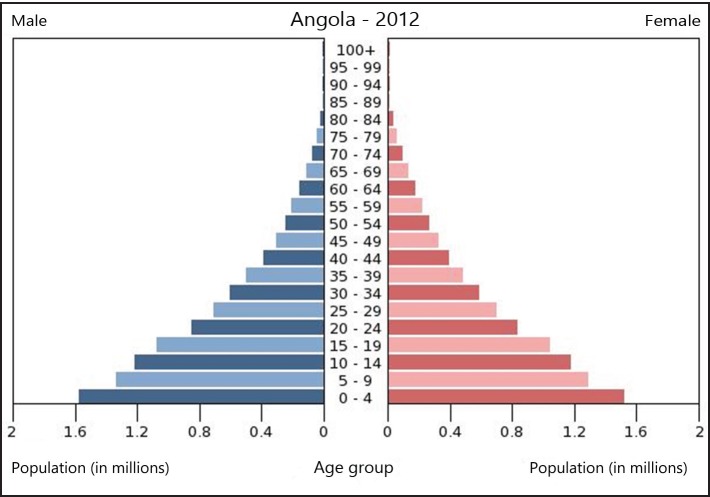
Angola’s population pyramid in 2012 [7].

In 2012, the Angolan population was estimated at 20 million inhabitants, a third of which live in the capital city, Luanda. Additionally, the country had a predominantly young population structure (Figure 1) and life expectancy was 50 years, 48 for men and 52 for women [7, 8].

Therefore, due to lack of a PBCR in Angola, the present study aimed to estimate the epidemiological profile of cancer in the country. This was performed by analysing the medical records of patients in the NOC, the only specialized hospital in the country, and the place to which patients with cancer in Angola are referred.

## Methods

The present study had a cross-sectional historical design and consisted of an investigation of the epidemiology of cancer in Angola through data from medical records of the NOC, the only Angolan hospital to specialize in cancer diagnosis and treatment.

The records of all patients referred to the NOC between 2007 and 2011 were reviewed. Cancer cases were defined as all patients diagnosed by clinical, radiological, histological, cytological, or haematological exams, on their first referral to the NOC, regardless of when the diagnosis was made.

In the first part of the study, cases between 2007 and 2011 were reviewed to investigate the location of tumours and determine the frequency of different types of cancer over the course of five years. These data were obtained from the NOC registration form, which contains data pertaining to new cases and is also used to assign identification numbers to each patient. Records from the year 2011 collected from hospital medical records were used to analyse the following variables: patient age, sex, tumour location, the type of diagnostic test conducted, cancer stage, source of patient referral, and patient address. Data from 2007 to 2010 were not used in this analysis because nearly 50% of medical records from this period were not available.

Data were collected by the main researcher between January and February 2012, and data collection was authorized by the NOC board of directors. Additionally, the present study was approved by the Research Ethics Committee of the Universidade Federal do Rio Grande do Sul. The study did not pose any risks to participants, and as secondary data were used, the study is not based on the informed consent. Patients remained anonymous, and all data were kept confidential.

Data were analysed using the Statistical Package for the Social Science (SPSS), version 18, and results were presented as absolute and relative frequencies in the form of graphs and tables. The means and standard deviations for the age of patients with different types of cancer were also calculated.

## Results

Between 2007 and 2011, the NOC detected 4,791 new cases of cancer, at an average annual incidence of 958 new cases. A 35% increase in cancer incidence was observed between 2007 and 2009 but decreased by 17% after this period. Over the course of the 5-year study period, the most common types of cancer diagnosed in patients of both genders were breast (20.5%), cervical cancer (16.5%), and head and neck cancers (10.6%), followed by lymphomas (7.2%), Kaposi sarcomas (6.1%), and prostate cancer (4%) (Table 1).

The incidence of most types of cancer appeared to wax and wane over the course of study period. However, the prevalence of prostate cancer increased steadily over the five years sampled, showing a 392% increase from 14 cases in 2007 to 69 cases in 2011 (Table 1).

**Figure 2.**
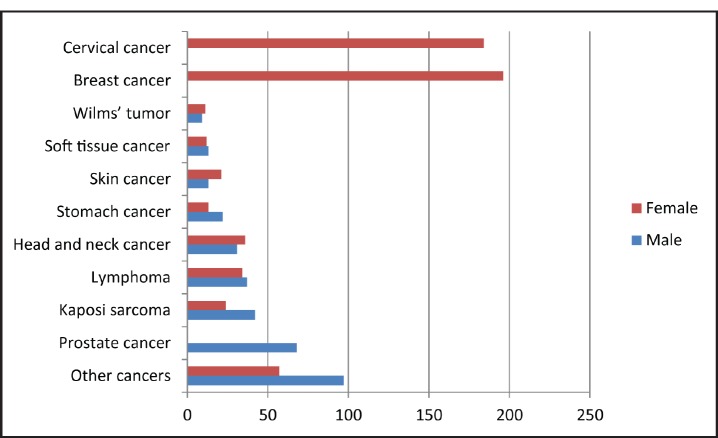
Diagnostic frequency of the 10 most common cancers in male and female patients treated at the National Oncology Centre of Luanda in 2011.

Medical records from the year 2011 were then reviewed for variables other than tumour location. It was found that most NOC patients were women (63.9%) and that the female to male ratio in cancer incidence was 1.7:1. The most frequently diagnosed cancers in women were breast and cervical cancer, while most male cancer patients were diagnosed with prostate cancer, followed by Kaposi sarcoma (Figure 2).

**Figure 3.**
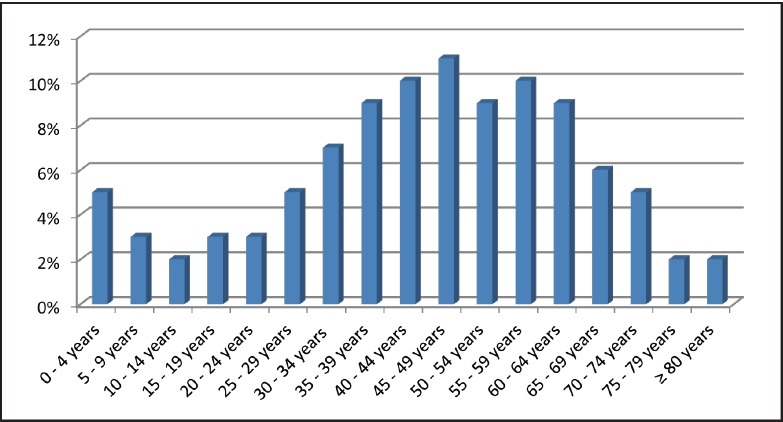
Age distribution of the 10 most frequently diagnosed cancers in the National Oncology Cancer of Luanda in 2011.

The analysis of age distributions showed that 76% of patients with cancer were younger than 60 years (Figure 3). The mean age of patients diagnosed with the most frequent cancers, which accounted for 51% of all cases in the sample, was also calculated. It was found that the mean age of patients with breast cancer was 46.7 years (± 14.4), while the mean age of patients with cervical cancer was 49 years (± 12.2). Patients with prostate cancer had a mean age of 66 years (± 9.2). However, when age groups were analysed separately, it was found that 32.4% of breast cancer cases were diagnosed in patients younger than 40 years, and 2.2% of cervical cancer cases occurred in women younger than 25 years (Table 2).

**Figure 4.**
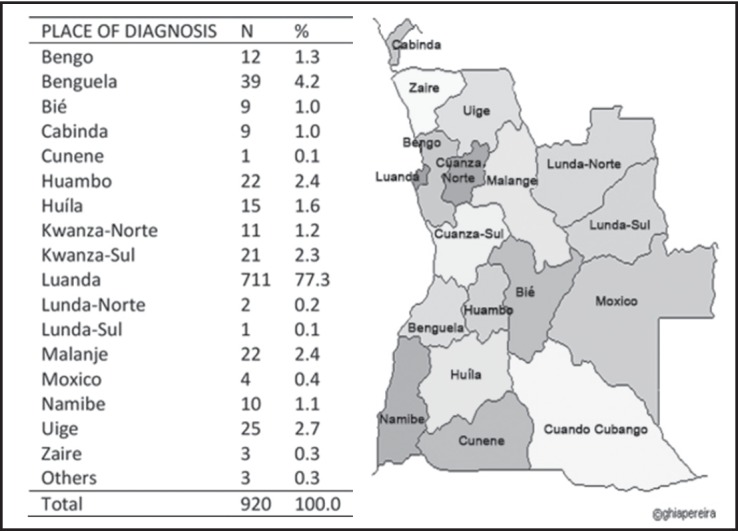
Distribution according to the province residency of patients diagnosed with cancer treated at the National Oncology Centre of Luanda in 2011.

As for patients’ place of residence, 77.3% of patients lived in Luanda, the capital of Angola, the place where the NOC is located. The Benguela and Uige provinces were the second and third most represented regions, accounting for 4.2 and 2.7% of patients, respectively. The remaining participants were from other provinces in the country (Figure 4).

Cancer staging was only analysed in patients with breast and cervical cancer. These data were not available for individuals with most other types of cancer. The analysis showed that most patients with these two types of cancer who sought treatment at the NOC were in advanced stages of the disease (Table 3).

## Discussion

Knowledge of the epidemiology of cancer in a given region or population is essential for public health planning, and an important tool for monitoring and evaluating cancer prevention and control programmes [4, 5].

The epidemiology of cancer is not well known in developing countries such as Angola, as these regions have no PBCR systems, and therefore, no sources of epidemiological data are found. In the absence of a PBCR in Angola, the present study aimed to describe the epidemiological profile of cancer in the country through the medical records of the NOC.

In 1987, an attempt was made to establish a National Cancer Registry in the country, but its existence was short-lived. However, the initiative did result in the publication of a series of cases registered during its 4-year operation period (1987 to 1990) until its discontinuation in 1991 [9, 10]. The report indicated that 1,552 new cases of cancer were identified in that period, at an average annual incidence of 388 new cases. The data also showed that 99% of patients referred to the NOC lived in the city of Luanda. Although the registry was discontinued, these data were later used by the IARC to estimate cancer incidence in Angola and in 2008, the institution estimated an incidence of 9,198 new cases in the country [11].

A comparison between the results from 1991 and those obtained in the present study revealed a 147% increase in the mean annual incidence of cancer. This could be attributed to an increase in the population of Angola (although there is no census in the country, the World Bank estimates that the Angolan population went from 15,417,943 in 2003 to 19,618,432 in 2011 [8]), renovations in the NOC, and a new hospital advertising campaign.

The end of the armed conflict in the country also allowed for the free movement of people and goods throughout the country, which lead to increased access to the NOC by patients from other provinces. However, the mean annual number of new cases detected in the present study corresponded to only 10% of the amount estimated by the IARC [11]. This discrepancy may result from the fact that our findings come from the only Angolan hospital to specialize in cancer treatment, since the country has no PBCR, while IARC data are population-based estimates for the entire country.

Therefore, we provided information on the cases that were diagnosed and referred to the NOC, although we believe that many cases are not referred to the hospital, especially those from rural areas. Additionally, some patients seen in the hospital may have been misdiagnosed or not diagnosed because of shortage of diagnostic resources and trained staff, and other accurately diagnosed patients are not treated because of practical and cultural issues.

Over the time period sampled in the present study, the most commonly diagnosed cancers, in descending order of frequency, were the following: breast, cervical, head and neck, lymphoma, Kaposi sarcoma, and prostate cancer. Similarly, high frequencies of breast and cervical cancer have been reported in other studies in the literature, many of which have also found that breast cancer (followed closely by cervical cancer) is the most common malignancy among women in developing countries [2, 11, 12]. The frequency of prostate and lung cancer in the present sample was lower than that of breast and cervical cancer. These findings are also in agreement with other studies conducted in African countries [12]. However, they differ from the results of epidemiological studies in countries such as the United States and Brazil, which report a much higher incidence of these cancers [13, 14].

The lower frequency of prostate cancer in the NOC as compared to breast and cervical cancer could be attributed to the fact that, at the time of data collection (January and February 2012), the NOC did not have a specialized in urology unit, and no radiotherapy clinics existed in Angola. Therefore, most patients with prostate cancer were treated in general hospitals, or in the Américo Boa Hospital, which has a urology service. These patients were only referred to the NOC when hormone therapy was required. The low life expectancy at birth in Angola (42 years in 2002 and 50 years in 2009) [15] may also have influenced results, as many patients may not reach the age brackets in which a higher prevalence of prostate cancer is observed. In developed countries, where life expectancy is over 70 years, studies report a higher prevalence of this cancer type. However, in developed countries such as the United States, the high incidence of prostate cancer may also be attributed to the implementation of prostate-specific antigen (PSA) screening programmes [16]. The low incidence of lung cancer found in the present study could be explained by its low frequency in the country or by difficulties involved in its diagnosis and referral.

The present results also showed that Kaposi sarcoma was the fifth most frequently reported cancer. This condition is often associated with AIDS [16]. The WHO has reported a high incidence and prevalence of AIDS in the African continent and has found that approximately 70% of AIDS patients in the world live in African countries. Although the prevalence of AIDS in Angola is relatively low compared to other African regions (2%), it is possible that the frequency of this condition is responsible for the higher rates of Kaposi sarcoma in the population [17]. Although this analysis was not initially planned in the study, an investigation of the medical records of patients diagnosed with Kaposi sarcoma found that 100% of these individuals tested positive for HIV. In countries with a high prevalence of AIDS, such as Zimbabwe, Kaposi sarcoma is a leading cause of cancer among black males and accounts for 23% of all cancer cases in men.

An analysis of general tendencies throughout the five years studied revealed a significant increase in the frequency of cancer cases between 2007 and 2009, followed by a decrease in subsequent years. The initial increase observed could be a result of the NOC advertising programme, which consisted of health talks and awareness campaigns for the community developed by its board of directors. The reduction in new cases of cancer in the fourth and fifth years studied could be due to the fact that the NOC began to require that general hospitals only refer patients with a confirmed diagnosis of cancer, which was previously not the case, as patients were often referred to the NOC for a diagnosis.

Staging data for breast and cervical cancer were also analysed. Most of these cases were diagnosed in late stages, which is a common finding in developing countries due to absent or ineffective cancer screening programmes [2]. In developed countries such as the United States, 75% of patients with cervical cancer and 60% of those with breast cancer are diagnosed in early stages [18].

Information about the distribution of cancer by site is also important for the planning of cancer prevention and control programmes, as it allows for the identification of the locations which would most benefit from the implementation of such interventions. The present findings indicated that 77% of patients with cancer in Angola live in the capital city of Luanda, which is also home to approximately one-third of the population of the country [19]. The fact that most of the patients studied lived in Luanda could be explained by the fact that the CNO is also located in the Angolan capital, allowing these patients to have easy access to the hospital. These results represent a marked change from the 1991 study, which had found that 99% of patients with cancer in Angola lived in Luanda [11]. It is possible that the significant increase in the number of patients from other provinces who travel to the NOC for treatment may be associated with the end of the armed conflict in the country, which had imposed restrictions on the free movement of people and goods within Angola.

The analysis of the age distribution of breast and cervical cancer helped to estimate the frequency of these conditions and to identify target groups for screening. The most commonly recommended screening test for breast cancer is the mammography, while the Pap smear is indicated for screening for cervical cancer [2].

Based on the morphophysiological characteristics of the breast, women are recommended to undergo mammography screenings starting at the age of 40 years. However, the greatest impact of mammography screening on mortality due to breast cancer occurs from 50 years of age onward, and certain high-risk groups are recommended to have regular screenings starting at the age of 40 [20, 22]. Conversely, in developing countries, due to lack of resources, the IARC recommends physical examination as the most appropriate screening method.

The present results revealed that 32.7% of patients with breast cancer were diagnosed before the age of 40 years. In a country with a life expectancy of 50 years, large proportion of breast cancer cases (and other types of cancer) are likely to develop in the young population, due to the low proportion of the population aged above 50 years, which have greater incidence rates of the disease. Within this perspective, the high percentage of breast cancer cases in women below 40 years found in our study are a matter of proportions in relation to incidence rates, since the proportion seems high because the incidence in general is very low [2].

Data regarding the prevalence of cervical cancer are also helped to define target groups for screening. As there is low risk of cervical cancer in women under 25 years of age (≤1%), health guidelines in many countries recommended that screening should start at the age of 25 [2, 23]. The present results showed an incidence rate of 2.2% in women under 25 years, which may be an important factor in deciding the age at which women should start undergoing regular screenings for cervical cancer.

## Conclusions

The present study aimed to evaluate the epidemiological profile of patients with cancer of the Angola NOC. The results indicated that breast and cervical cancer were the most common malignancies in female patients, while prostate cancer was the most common form of cancer in male patients. However, the incidence of prostate cancer was much lower than that of breast or cervical cancer. Although staging data were not available for most types of cancer, the patients for whom this variable was available appeared to have been diagnosed in advanced stages of cancer. Analyses of the age distribution of patients with breast cancer showed that a significant percentage of these individuals were diagnosed prior to the age of 40 years.

The present results must be interpreted in the light of following limitations:

- During the study period, all cases of cancer are not detected by this study, as some individuals (especially those in rural areas) do not obtain treatment at health care facilities.
- The hospital-based design prevented the inclusion of patients who were diagnosed in other hospitals and not referred to the NOC. Although it is the only specialized cancer hospital in Angola, patients who lived far from the NOC may not have been referred to this centre for practical reasons and their profile may be different from that described by our results.
- The shortage of human and technological resources may limit the hospital’s ability to diagnose cancer even in patients who do seek treatment at health care facility.

In spite of these limitations, it is extremely likely that most patients with cancer in Angola are referred to the NOC for treatment, since it is the only specialized cancer hospital in the country, so that the present results constitute a reasonable estimate of epidemiological patterns of cancer in Angola, although it corresponded to only a small percentage of the number of new cases estimated by the IARC. However, the implementation of a well-controlled and comprehensive PBCR in the country is strongly recommended, so that even more comprehensive and accurate data regarding the epidemiology of cancer in Angola could be obtained.

## Financial support for manuscript translation

National Oncology Centre (NOC).

## Translation assistance

Scientific Linguagem Ltda.

## Conflicts of interest

The authors declare that they have no conflict of interest.

## Authors’ contributions

In the conception and design of the study, acquisition and analysis of data, and writing the manuscript, António Armando provided his support. Mary Clarisse Bozzetti was involved in the conception and design of the study and writing the manuscript. Alice de Medeiros Zelmanowicz contributed to the conception and design of the study, data analysis, and writing of the manuscript.

## Figures and Tables

**Table 1.** The 10 most common cancers in the National Oncology Centre of Luanda from 2007 to 2011.

Diagnosis	Years					Total	%
	2007	2008	2009	2010	2011		
Breast cancer	199	164	212	210	196	981	20.5
Cervical cancer	112	143	200	159	184	798	16.7
Head and neck cancer	86	124	146	124	67	547	11.4
Lymphoma	48	66	75	68	71	328	6.8
Kaposi Sarcoma	55	62	50	63	66	296	6.2
Skin cancer	45	47	58	50	34	234	4.9
Prostate cancer	14	17	31	55	69	186	3.9
Stomach cancer	24	21	31	25	35	136	2.8
Lung and pleural cancer	18	22	23	15	18	96	2.0
Wilms’ tumour	8	10	20	22	20	80	1.7
Other cancers	204	286	256	203	160	1109	23.1
Total	813	962	1102	994	920	4791	100.0

**Table 2.** Age distribution of patients diagnosed with breast, endometrial, and prostate cancer at the National Oncology Centre of Luanda in 2011.

Age group	Cervical cancer		Breast cancer		Prostate cancer	
	%	CP	%	CP	%	CP
≤ 20 years	1.1	1.1	2.0	2.0	0.0	0.0
20–24 years	1.1	2.2	2.6	4.6	0.0	0.0
25–29 years	1.6	3.8	5.1	9.7	0.0	0.0
30–34 years	5.9	9.7	7.7	17.3	0.0	0.0
35–39 years	9.1	18.8	15.3	32.7	0.0	0.0
40–44 years	17.2	36.0	17.3	50.0	0.0	0.0
45–49 years	19.9	55.9	11.2	61.2	1.5	1.5
50–54 years	11.8	67.7	10.2	71.4	10.3	11.8
55–59 years	10.8	78.5	10.7	82.1	10.3	22.1
60–64 years	10.8	89.2	6.6	88.8	23.5	45.6
65–69 years	5.4	94.6	3.6	92.3	14.7	60.3
70–74 years	3.2	97.8	4.1	96.4	20.6	80.9
≥ 75 years	2.2	100.0	3.6	100.0	19.1	100.0
Total	100.0		100.0		100.0	

CP: cumulative percentage

**Table 3.** Stage distribution of breast and cervical cancer among patients treated at the National Oncology Centre of Luanda in 2011.

Stage	Location			
	Breast cancer		Cervical cancer	
	Number of cases	%	Number of cases	%
0	1	0.5	4	2.2
I	5	2.5	3	1.6
II	20	10.2	31	16.8
III	120	60.9	96	52.2
IV	17	8.6	13	7
No staging data available	33	17.3	37	20.1
Total	196	100	184	100
